# Analysis of the Influence of Breastfeeding and Bottle-Feeding upon the Origin of Posterior Crossbites

**DOI:** 10.3390/children11020182

**Published:** 2024-02-02

**Authors:** Antonio Francisco Galán-González, Antonia Domínguez-Reyes, Inés María Marín-Castro, Lourdes Muñoz-Muñoz, María Eugenia Cabrera-Domínguez

**Affiliations:** Department of Stomatology, University of Seville, 41009 Seville, Spain; agalan@us.es (A.F.G.-G.); inesmmarin@us.es (I.M.M.-C.); lmm@us.es (L.M.-M.); mcabrera@us.es (M.E.C.-D.)

**Keywords:** breastfeeding, bottle-feeding, malocclusion, posterior crossbite, deciduous dentition

## Abstract

(1) Introduction. An analysis was made of posterior crossbites in deciduous dentition and their relation to the type of feeding received by the child, with the objective of determining the influence of the way in which the child is fed in the early stages of life on the development of posterior crossbites. (2) Material and methods. A total of 1401 preschool children between 3 and 6 years of age from Seville (Spain) were included in the study. An intraoral exploration was carried out to assess the presence of crossbites (uni- or bilateral, and functional or not). The study was completed with a parent or legal guardian questionnaire exploring the type of feeding received by the child in the first stages of life, as well as the presence of bad oral habits and their duration. (3) Results. A total of 276 children (19.7%) presented posterior crossbite in occlusion. Uponn centering the midlines, 197 were maintained, indicating that 79 were due to premature contacts (functional crossbites). There were no significant differences in crossbites among the children who had received breastfeeding, though bottle-feeding was seen to favor crossbite. (4) Conclusions. No statistically significant relationship was found between posterior crossbites and breastfeeding, though an association between posterior crossbites and bottle-feeding was observed, with the number of crossbites increasing with the duration of bottle-feeding.

## 1. Introduction

Upon completion of the eruption of the primary dentition at approximately 30 months of age, the opposing teeth come into contact with each other, i.e., occlusion takes place. Occlusion is defined as the relationship between the antagonistic teeth and the dental arches in a plane known as the occlusal plane. At present, this concept encompasses a more dynamic perspective, including not only the teeth and the dental arches but also the covering tissues; the adjacent muscles; the morphology and function of the temporomandibular joints (TMJs); the different functions of the stomatognathic system (chewing, swallowing, speech, and breathing); and the neuromuscular, neuro-occlusal, and postural factors of the individual [1,2,3].

Normal occlusion in the primary dentition is considered to be when there are interincisive diastemas and primate spaces; a slight overbite and overjet; a straight terminal plane; a Class I molar and canine relationship; upper and lower incisors with an almost vertical inclination, i.e., a very open interincisor angle; and, transversely, when the upper vestibular cusps overlap the lower vestibular cusps.

Malocclusion is considered to occur when there are no diastemas or primate spaces; when there is more than a two-thirds underbite, an open bite, or a pronounced overbite; when there is a distal terminal plane or long mesial; Class II or III molar and canine relationship; a closed interincisal angle; and, transversely, a crossbite.

The occlusion of the primary dentition is influenced by a number of characteristics which, while not decisive, can be considered favorable signs of correct future occlusion in the adult final dentition. One of these conditioning characteristics is that, buccolingually, the upper teeth must surpass the lower teeth, with the internal cusps of the maxillary molars occluding in the anteroposterior sulcus that separates the external cusps from the internal cusps of the lower molars. When this condition is not met, and the lower molars surpass the maxillary molars, the situation is referred to as crossbite [4].

Malocclusion is one of the most common pathologies in the oral cavity, and posterior crossbites are one of the most common, with a prevalence of between 0.3 and 42.5% [2,5,6,7,8]. These are the reported extreme values, and although they may suggest a great disparity of results among different authors, the fact is that apart from some exceptions, our review of the literature evidenced great uniformity among the studies as regards the prevalence of posterior crossbite.

Crossbite is usually caused by more than one etiological factor, and the influencing conditions may range from a simple environmental cause to complex interactions between the individual’s genetics and the environmental factors to which they are exposed. It is quite possible that malocclusions are the result of both factors, probably because they are the result of a genetically determined occlusion, in turn worsened by functional forces, feeding, or parafunctions that may have an impact on the growth of the individual [9]. Reciprocally, genetically disadvantageous conditions could be positively altered by the influence of environmental factors during growth. It can, therefore, be said that the etiological factors responsible for malocclusions in deciduous dentition comprise both intrinsic factors that condition development and external or environmental factors [2,3,5,9].

In relation to the environmental factors, mention can be made of the feeding received by the child in the first few months or years of life, mainly breastfeeding or bottle-feeding, with the former traditionally being considered to act as a beneficial factor in the development of the orofacial structures [10,11,12], while bottle-feeding has been held to be a suction feeding habit that proves harmful for the development of these structures [11,13,14,15]. However, there is no clear agreement among authors regarding these associations, and the role of maternal breastfeeding (duration and exclusiveness) in relation to normal occlusion or of bottle-feeding in relation to oral malocclusions is not clear [16,17,18,19]. The published systematic reviews in this field yielded contradictory results [14,17,19,20,21]. As commented above, there is considerable agreement in the number of posterior crossbites found, though discrepancies are observed when it comes to the possible repercussions of feeding in the first years of life, i.e., breastfeeding, bottle-feeding, or mixed-feeding, upon the origin of posterior crossbite.

There is currently no doubt about the importance of breastfeeding for the psychological, physical, and immunological development of the child [21,22], though its influence upon the development of the maxillofacial system has not been fully defined [17,20].

After reviewing and contrasting different articles and meta-analyses on the oral repercussions of the type of feeding in the first stages of life, we found that the results of the different authors vary considerably. Furthermore, although we know that the origin of crossbite is influenced by more than just one factor, as commented above, it is known that certain extrinsic factors can favor malocclusion, and bottle-feeding, for a prolonged period of time, may be one of them.

The authors considered it to be very important to carry out a study in our setting on the oral repercussions of breastfeeding and bottle-feeding, especially in relation to the origin of crossbites, for two main reasons: (a) the high prevalence of crossbite; (b) because detection of the factors causing crossbites would make it easy to prevent and even treat them if detected in an early stage, using cost-effective treatments that afford very satisfactory outcomes. Therefore, the aim of our study is to know the possible influences of breastfeeding or bottle-feeding on the genesis of posterior crossbites in the infant population of Seville.

## 2. Materials and Methods

This study was carried out during the 2021–2022 academic year.

Sample size calculation was carried out using the GPower 3.1 application. In order to obtain a sufficiently representative sample, we randomly selected three schools in each of the districts that make up Seville (Spain), six in total—one for every economic level (high, middle, and low). This ensured a random sample, as there was no preselection or bias. The following inclusion criteria were applied:

- Aged between 3 and 6 years, inclusive.
- Enrollment in one of the chosen schools.
- Parents who had completed the questionnaire.
- Do not have genetic or systemic changes (maxillary hypoplasia, mandibular hyperplasia, malformation syndromes, etc.) that could influence the conclusions of the study.
- No past or present orthodontic treatment of any kind.
- No harmful oral habits (dummy, digit sucking) persisting long enough to permanently alter dental occlusion. Dummy use for over two years was considered to be bad for occlusion, in the same way as digit sucking for over one year, due to the increased force exerted by the digit in the mouth.
- Informed consent from the parents or legal guardians.

All the children that met the study inclusion criteria were explored by a single dentist with over 10 years of professional experience to determine the presence or absence of uni- or bilateral crossbites in occlusion. The crossbites were diagnosed by direct clinical inspection. Those children with crossbites also underwent a functional analysis, taking the mandible to the resting position and checking the alignment of the interincisal frenula and midlines to discard crossbites as a result of early contact and establish the type of crossbite. This analysis was not reflected in practically any of the consulted publications. We feel it to be of great importance and an innovation of the present study, since it allowed us to discard crossbites as a result of early contacts, which are often not skeletal crossbites that may be treated in a simple manner by trimming the contacts.

The explorations were carried out using the materials commonly used in epidemiological studies on oral health: intraoral mirrors, dental probes, tongue depressors, antiseptic solution, containers for material, gloves, masks, and paper towels.

To carry out the study, the child was seated and well-positioned to ensure full visibility during exploration of the oral cavity under good natural light complemented by a direct artificial light source. We first assessed dental occlusion from the frontal and right and left lateral angles and in maximum intercuspidation—analyzing the presence or absence of posterior crossbites. After diagnosing posterior crossbite, the mandible was gently manipulated to secure deocclusion, checking alignment of the midlines. At this point and without dental contacts, we again analyzed the presence or absence of posterior crossbite. In this context, we could observe crossbite in the same way as in occlusion, or variation towards crossbite of the contralateral side, bilateral crossbite, or no crossbite. In the latter case, the child was instructed to bite down slowly in order to identify possible premature contacts as the cause of functional crossbite.

After the completion of the oral examination, a questionnaire was sent to the parents to assess the feeding received by the child in the first months of life (breastfeeding or bottle-feeding) and any present or previous harmful habits, the frequency with which they occur, and how long they lasted. The parents completed the questionnaire in accordance with the instructions and under the supervision of qualified personnel. Each harmful habit of the mouth was clarified by the examiners, and the participants’ questions were answered satisfactorily.

### 2.1. Ethical Approval and Consent to Participation

The parents were informed of the objectives of the research. Written informed consent was obtained from all participants. The study protocol abided by the recommendations of the Declaration of Helsinki on research in human subjects and was approved by the Biomedical Research Ethics Committee of the Andalusian Government (Ref. code 0937-N-15).

### 2.2. Statistical Analysis

A general descriptive analysis of the children was made, with cross-comparisons of the qualitative variables. The chi-square test was applied to identify parameters with significant differences, using Haberman-adjusted residues, to independently determine the significance of the differences in percentage values with respect to the total sample.

The data were entered in MS Excel 2021 spreadsheets, and the SPSS version 26 statistical package for Windows (IBM Corp., Armonk, NY, USA) was used for analysis. Statistical significance was considered for *p* < 0.05.

## 3. Results

A total of 1723 preschool children were selected, of which only 1401 (776 girls and 625 boys) met all the inclusion criteria. The age distribution was as follows: 3-year-olds, 53 children; 4-year-olds, 501; 5-year-olds, 690; and 6-year-olds, 157.

Crossbites were studied both in occlusion and in functional testing, aligning the two interincisal midlines. It was seen that in occlusion, 1125 children (80.3%) presented normal occlusion, i.e., they showed no posterior crossbite, while 276 (19.7%) presented crossbite (Table 1).

Right crossbites were significantly more frequent in girls than in boys (*p* < 0.05), though such statistical significance was not observed for the rest of the crossbites.

On centering the two midlines (upper and lower) to exclude crossbites due to premature contacts (functional analysis), we found that of the 276 children with posterior crossbite in occlusion only 197 continued to present crossbite, i.e., in 79 cases (5.6% of the total sample) crossbite was attributable to premature contacts. This is what is defined as functional crossbite.

On excluding the 79 cases corresponding to functional crossbites, the skeletal crossbites are shown in Table 2. While a slight female predominance persisted, statistical significance was not reached in this case.

Since crossbites are often attributed to environmental factors, and the purpose of our study was to analyze the role of the type of feeding (breastfeeding or bottle-feeding) in the origin of crossbites, we explored the possible existence of statistically significant associations between the feeding received by the child and the development of crossbite.

We were interested in determining how many children had initially received breastfeeding and for how long; how many simultaneously or consecutively received breastfeeding and bottle-feeding; and how many had received only bottle-feeding up until the time of switching to spoon-feeding.

Taking into account that breastfeeding is considered to favor good development of the dental arches, we found that 81.3% of our sample (1139 children) were initially breastfed, while 18.7% (262 children) were never breastfed and always received bottle-feeding (Table 3).

Breastfeeding and direct progression to spoon-feeding (e.g., paps) was recorded in 147 children (10.5%), with combined breastfeeding and bottle-feeding in 992 children (70.8%).

**Figure 1:**
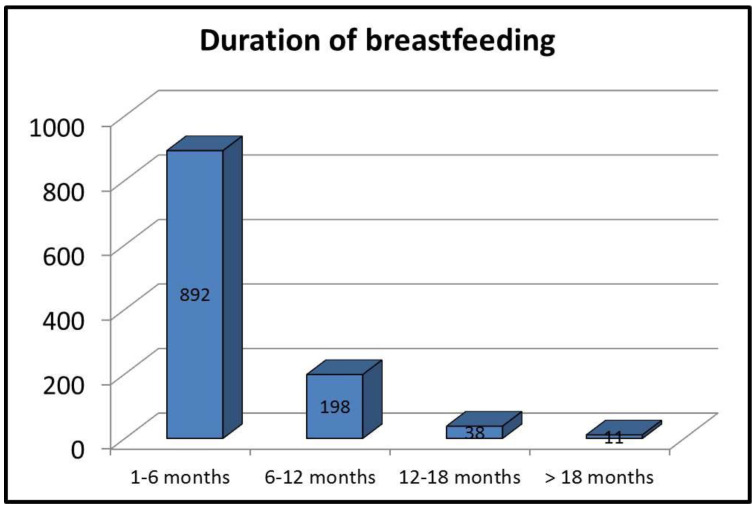
Duration of breastfeeding.

The duration of breastfeeding is reflected in Figure 1.

Whether combined with breastfeeding or not, and whether given for some time or still maintained at the time of the study, we found that 1255 children (89.6% of the sample) had received bottle-feeding at some point during development, while 10.4% (146 children) had never been bottle-fed (Table 4).

**Figure 2:**
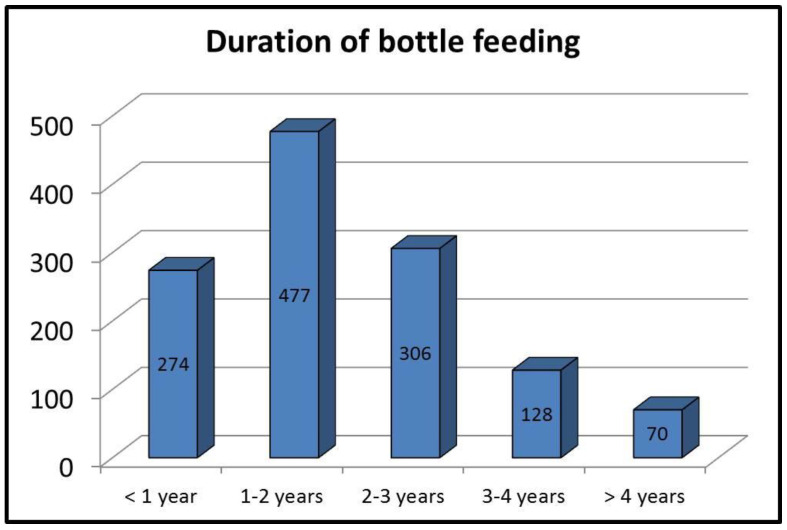
Duration of bottle-feeding.

Of the children that were bottle-fed, 263 (20.9%) received this type of feeding only, while 992 children (79.1%) received a combination of bottle-feeding and breastfeeding. The duration of bottle-feeding is shown in Figure 2.

The statistical analysis of the relationship between crossbites and the type of feeding showed no correlation between breastfeeding or its duration and the development of crossbites.

In contrast, a significant relationship was found between the duration of bottle-feeding and crossbites, though only when the latter were considered after the functional analysis and on the right side. Specifically, the longer the duration of bottle-feeding, the greater the tendency to develop crossbite (Table 5).

## 4. Discussion

Our results show a statistically significant association between bottle-feeding and the occurrence of subsequent crossbites, but not when breastfeeding is taken into account.

All of the studies that were consulted used a methodology that is similar to the one used in this study: examination of the child’s dental occlusion under direct clinical vision and a questionnaire for parents or guardians. In some studies, the questionnaire was a substitute for a face-to-face interview with parents or guardians [5,23,24,25,26].

In terms of sample size, we found three papers with more than 2000 children studied [2,9,24]. A total of five studies included fewer than 500 children [5,10,25,26,27]. With sample sizes similar to ours, we found the works of Kobayashi [28], Paolantonio [23], Romero [29], and Viggiano [30].

The type of study was mostly cross-sectional epidemiological, like the one we conducted. However, in five cases, longitudinal studies were conducted [2,5,7,31,32].

Many studies were made on the occlusion and malocclusion in permanent dentition, though few studies analyzed occlusion in deciduous dentition, and even fewer assessed the repercussions of the type of feeding in the first stages of life on the risk of developing malocclusion.

On the other hand, with regard to the detection of possible functional crossbites, we identified only one analysis, published by Kobayashi [28], who recorded a 9.4% incidence of functional crossbites versus 5.6% in our own study. As a result, in relation to the rest of the consulted literature, we had to establish a comparison with results that referred to crossbites in occlusion.

Regarding unilateral crossbites, the 16.6% incidence recorded in our study is very similar to the 15% reported by Paolantonio [23] and the 14% incidence recorded by Ovsenik [33] and Dimberg [7] in 7-year-old children. Dimberg observed a 13% incidence of unilateral posterior crossbites in 3-year-old children.

Figures lower than our own were published by Duncan [31] (10.5%), Bandeira [24] (7.02%), Kobayashi [28] (4.4%), and Wagner [27] (3.4%).

With regard to bilateral crossbites, the percentage recorded in our sample was 3.1%, which is consistent with the data published by Dimberg [7] in 7-year-old children (3.0%) and reported by Kobayashi [28] (2.8%). Comparatively lower figures were obtained by Duncan [31] (2.2%), Bandeira [24] (1.78%), and Ovsenik [33] (1.2%) in 5-year-old children from Slovenia. We only documented incidences higher than our own in the article published by Dimberg [7] but in 7-year-old children (6.0%). In France, Tschill [34] reported that bilateral crossbites are observed in 2.4% of girls between 4 and 6 years of age and in 5.2% of boys of the same age.

Overall, the total number of posterior crossbites, whether uni- or bilateral, was one of the least discordant parameters we found, with the exception of the 42.5% incidence reported by Saliba Moimaz [5]. Specifically, and compared with our figure of 19.7%, very similar percentages were reported by Germa [32] (20.0%), Tschill [34] (19.6%), and Peres [25] (18.2%). Somewhat different prevalences were published by Osevnik [33] (15.2%), Limeira [35] (15.0%), Bandeira [24] (10.4%), Khda [36] (10.1%), Lopes-Freire [37] (8.1%), Zhou [2] (7.56%), and Kobayashi [28] (7.2%).

With regard to the type of feeding received by the children in the first stages of life, breastfeeding is widely acknowledged to favor normal development of the orofacial muscles since these must be exercised by the infant while feeding [29,38,39]. This led to the development of pro-breastfeeding campaigns, which we believe led to a greater number of children being breastfed at some point in their lives.

A number of authors, such as Peres [25], Chen [8], and Limeira [35], found a longer duration of breastfeeding to be significantly correlated to a lower probability of developing posterior crossbite. However, some investigators, such as Germa [32], observed no such correlation. Likewise, in our study, no statistically significant association was observed between the duration of breastfeeding and the development of posterior crossbites.

The above observations do not apply to bottle-feeding or artificial feeding, for, as pointed out by Viggiano [30], the suction mechanisms differ between breastfeeding and bottle-feeding. In this regard, different authors such as Warren [40] and Farsi [41] commented on the relationship between bottle-feeding and the appearance of malocclusions. In our study, there was a statistically significant association between the duration of bottle-feeding and the appearance of crossbites, though only when the latter were considered after the functional analysis and on the right side. In this regard, a longer duration of bottle-feeding correlated to a greater incidence of this type of crossbite. This relationship was also reported by authors such as Viggiano [30], Vázquez-Nava [42], and Moimaz [5], though it also must be noted that other investigators such as Lopes-Freire [26] and Chen [8] found no such association.

Overall, although we found few discrepancies among the different authors regarding the prevalence of posterior crossbites (considering only crossbites in occlusion, for, as mentioned above, only Kobayashi [28] considered the functional analysis used in our study), there was less agreement on the possible repercussions of breastfeeding or bottle-feeding on the origin of such crossbites. The reasons for this could include the type of study sample involved, possible sample bias, the socioeconomic level of the subjects, the presence of other habits that prove harmful for occlusion, possible previous orthodontic treatment, and the reliability of the questionnaires answered by the parents or legal guardians. We know that in epidemiological studies with a large sample size, factors altering randomness may be present, and although we tried to be very strict in this regard, partial sample bias cannot be ruled out in our study.

There are other limitations to this study. On considering the types of feeding, and although we excluded those children with bad oral habits persisting long enough to alter dental occlusion, there may have been certain habits (such as mouth breathing or immature swallowing) which the parents might not have been able to identify. Therefore, the influence of the type of feeding on the posterior crossbite may have been altered by this fact. In addition, the lack of published crossbite studies with functional analysis meant that we were unable to compare our results with those of other researchers. Likewise, although the parents received adequate information about the feeding received by their child in the first stages of life, there may have been some subjectiveness in the communication of the information required by the study.

The strengths of the study include the functional analysis of crossbite and the exclusion of parafunctional habits.

Despite the above, we consider that the type of feeding in the first stages of life plays an important role in the origin of posterior crossbites. Although some authors postulate that the spontaneous correction of these alterations occurs when the children progress from the deciduous dentition to the mixed or permanent dentition [7,36], the identification of such factors may contribute to the prevention of these problems, or, if crossbite has already become established, early treatment could avoid more complex and expensive orthodontic treatments in the future.

Further research in this line of epidemiological study, such as the one carried out by Kinzinger, would be necessary to prevent and predict the possible future need for orthodontic treatment in a population [43].

This study could be extended in the future with more precise studies using 3D cone beam study, while always justifying the effects of radiation on growing and developing patients [44].

## 5. Conclusions

We found that 276 children (19.7%) had crossbite in occlusion, and 1125 (80.3%) did not.

After functional analysis, it was observed that 197 maintained crossbite and that in 79 cases (5.6% of the total sample) it was attributable to premature contacts (functional crossbite).

A total of 1139 children (81.3%) had been breastfed at some point in their life, while 262 children (18.7% of the sample) had never been breastfed.

Of those breastfed, 147 (10.5%) switched directly to spoon feeding, and 992 children (70.8%) received a combination of breastfeeding and bottle-feeding.

In total, 1255 children (89.6%) were bottle-fed at some point, and 146 children were never bottle-fed. Of those who were bottle-fed, 263 (20.9%) were exclusively bottle-fed, while 992 children (79.1%) received a combination of bottle-feeding and breastfeeding.

We found no correlation between breastfeeding or its duration and the development of crossbites.

We did observe a statistically significant association between the duration of bottle-feeding and the occurrence of crossbites, although only in the crossbite considered after functional analysis and on the right side.

The longer the duration of bottle-feeding, the higher the probability of developing crossbite.

## Figures and Tables

**Table 1:** Crossbites in occlusion and gender distribution.

Crossbites in Occlusion	Boys	Girls	Total
No crossbite	519 (83.3%)	606 (77.9%)	1125 (80.3%)
Right crossbite	41 (6.5%)	82 (10.6%) **(*p* = 0.0160)**	123 (8.8%)
Left crossbite	44 (6.9%)	63 (8.2%)	107 (7.6%)
Bilateral crossbite	21 (3.3%)	25 (3.3%)	46 (3.3%)
**Total**	625	776	1401

**Table 2:** Crossbites (after functional analysis) and gender distribution.

Crossbites after Functional Analysis	Boys	Girls	Total
No crossbite	546 (87.4%)	656 (84.6%)	1203 (85.9%)
Right crossbite	23 (3.7%)	42 (5.4%)	65 (4.6%)
Left crossbite	23 (3.7%)	35 (4.5%)	57 (4.1%)
Bilateral crossbite	33 (5.2%)	43 (5.5%)	76 (5.4%)
**Total**	625	776	1401

**Table 3:** Distribution of breastfeeding by gender.

Breastfeeding	Boys	Girls	Total
Breastfeeding	515 (82.4%)	624 (80.4%)	1139 (81.3%)
No breastfeeding	110 (17.6%)	152 (19.6%)	262 (18.7%)
**Total**	625	776	1401

**Table 4:** Bottle-feeding. Gender distribution.

Bottle-Feeding	Boys	Girls	Total
Bottle-feeding	556 (89.0%)	698 (90.0%)	1255 (89.6%)
No bottle-feeding	69 (11.0%)	78 (10.0%)	146 (10.4%)
**Total**	625	776	1401

**Table 5:** Relationship between bottle-feeding and functional crossbite.

		Duration of Bottle-Feeding				
		Never	1–6 Months	7–12 Months	13–18 Months	>18 Months
**Right crossbite** ***p* = 0.025**	**No crossbite**	139 (9.9%)	153 (10.9%)	373 (26.6%)	156 (11.1%)	514 (36.7%)
	**Crossbite**	7 (0.5%)	3 (0.2%)	15 (1.1%)	9 (0.6%)	32 (2.3%)
**Left crossbite**	**No crossbite**	143 (10.2%)	149 (10.7%)	373 (26.6%)	161 (11.5%)	519 (37.0%)
	**Crossbite**	3 (0.2%)	7 (0.5%)	15 (1.1%)	4 (0.3%)	27 (1.9%)
**Bilateral crossbite**	**No crossbite**	142 (10.1%)	146 (10.4%)	365 (26.1%)	152 (11.0%)	520 (37.1%)
	**Crossbite**	4 (0.3%)	10 (0.7%)	23 (1.6%)	13 (0.9%)	26 (1.9%)

## Data Availability

The data presented in this study are available on request from the corresponding author. The data are not publicly available due to privacy and ethical restrictions.
